# The Effect of Physical Exercise on Patients With Mild Cognitive Impairment: A Scoping Review

**DOI:** 10.7759/cureus.73265

**Published:** 2024-11-08

**Authors:** Vasileios T Stavrou, George D Vavougios, Georgios M Hadjigeorgiou, Panagiotis Bargiotas

**Affiliations:** 1 Department of Neurology, Medical School, University of Cyprus, Nicosia, CYP; 2 Department of Respiratory Medicine, Laboratory of Cardio-Pulmonary Testing and Pulmonary Rehabilitation, Faculty of Medicine, University of Thessaly, Larissa, GRC

**Keywords:** biomarkers, evidence based, evidence-based, exercise, exercise benefits, exercise modes, health benefits, mci, mild cognitive impairment, neurological biomarkers

## Abstract

Mild cognitive impairment (MCI) is characterized by a noticeable decline in cognitive abilities that is not severe enough to significantly interfere with daily life or independent functioning. Recent research highlights the important role of exercise in managing and improving cognitive function in patients with MCI. This scoping review examines the benefits of different forms of exercise in improving cognitive function. Recommendations for exercise, including frequency, consistency, and individualized programs, are discussed in this review, with an emphasis on the importance of safety and regular monitoring. The integration of physical and cognitive training is also suggested to maximize benefits. Regular physical exercise is a promising intervention for mitigating cognitive decline and improving the overall quality of life in patients with MCI.

## Introduction and background

Physical exercise (PE) is any bodily activity that improves or maintains physical fitness, general health, and well-being. PE refers to the subset of planned and repetitive physical activities that are designed to improve physical fitness using skeletal muscle and require the expenditure of energy [1]. Exercise can include various forms of activity (e.g., walking, running, swimming, cycling, dancing, weight training, individual and/or team sports, etc.) and can be categorized into three main types as follows: (a) aerobic exercise, (b) resistance training, and (c) flexibility and balance exercises and combinations thereof. Aerobic exercise includes activities such as walking, cycling, etc., that increase heart rate and improve cardiorespiratory endurance at an intensity of approximately 50-70% of maximum heart rate or 40-60% of maximum oxygen uptake. Strength training, which focuses on building muscle strength and endurance, involves contracting your muscles against external resistance, such as your body weight, dumbbells, etc. Flexibility and balance exercises are activities (e.g., yoga, Pilates, etc.) that improve range of motion, flexibility, and balance to keep the body in a stable position when performing certain movements, improve coordination, and prevent falls [2].

**Figure 1:**
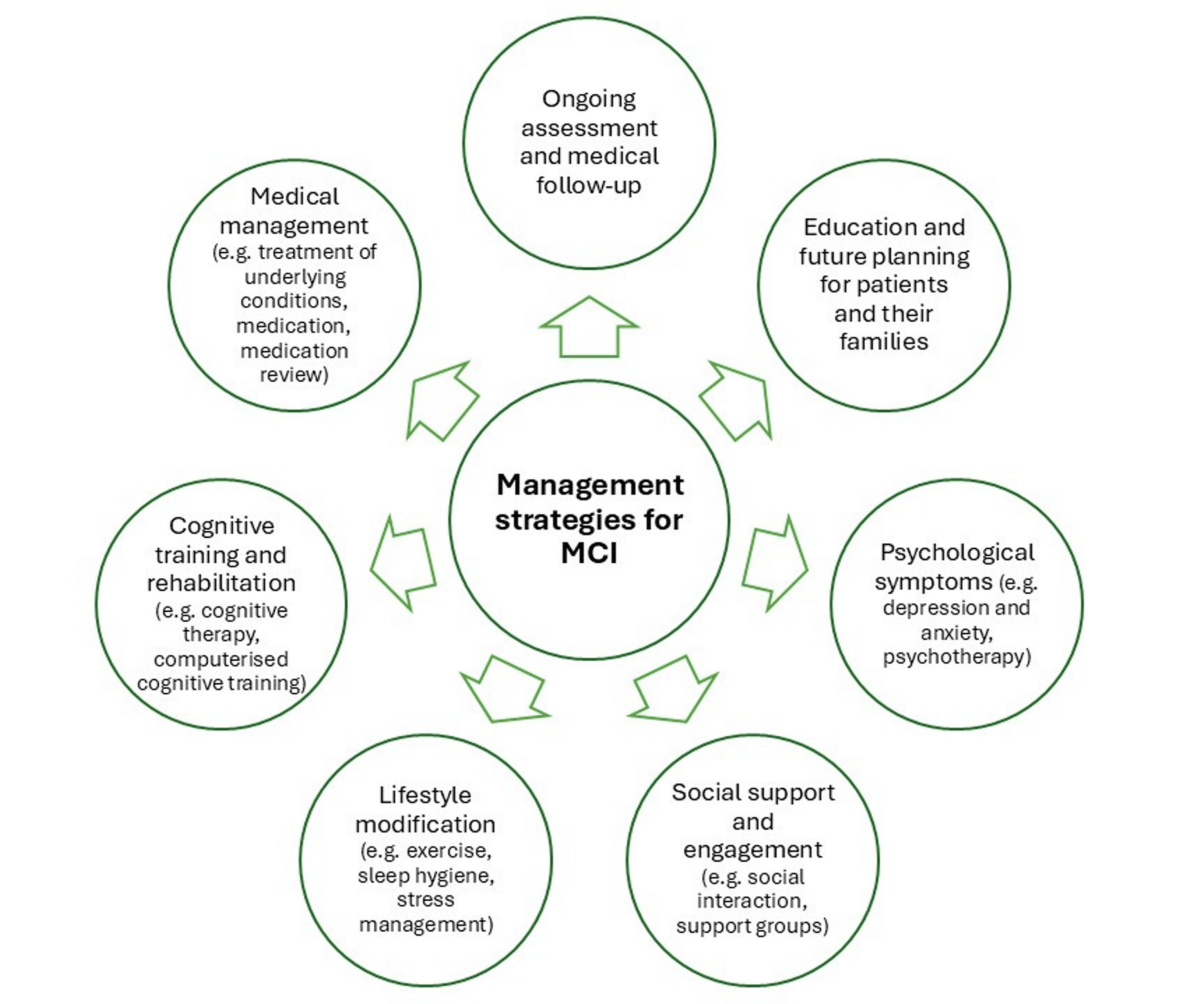
Indicative management strategies for mild cognitive impairment (MCI) - lifestyle change and symptom management strategies. The figure is designed by the author (Vasileios T. Stavrou) of this study.

Mild cognitive impairment (MCI) is a condition characterized by noticeable cognitive decline that is greater than expected for a person's age but does not significantly interfere with daily life or independent functioning (Figure 1). People with MCI have problems with memory, language, thinking, or judgment [3]. However, these problems are not severe enough to be classified as dementia. MCI may represent a transitional stage between normal cognitive aging and more serious conditions such as Alzheimer's disease. There are the following two main types of MCI: (i) amnestic MCI - this type primarily affects memory, people may often forget important information that they used to remember easily, such as appointments, conversations, or recent events; and (ii) non-amnestic MCI - this type affects cognitive functions other than memory, such as language, attention, or visual-spatial skills [4]. For example, a person may find it increasingly difficult to make decisions, carry out complex tasks, or recognize familiar objects. While MCI increases the risk of developing Alzheimer's disease or another type of dementia, not everyone with MCI will progress to these conditions. Some people may remain stable or even return to normal cognitive function over time. Diagnosis usually involves a combination of medical history, cognitive tests, neurological examinations, and sometimes brain imaging to rule out other causes of cognitive decline [5]. MCI has emerged as a major public health concern, lying at the critical intersection between normal aging and more severe forms of cognitive decline such as dementia. Understanding MCI is essential for both individuals and healthcare systems as it affects millions of people worldwide, particularly as populations age. While MCI does not always progress to more serious conditions such as Alzheimer's disease, it signals a change in cognitive function that can affect daily functioning and quality of life, making it a critical condition to recognize, study, and manage.

The prevalence of MCI is increasing with the rise of neurodegenerative diseases in the aging population. As life expectancy increases worldwide, the number of people living with cognitive impairment, including MCI, is expected to rise. According to the World Health Organization (WHO), the global population of people aged 60 years and over will double by 2050 [6]. This demographic shift presents a challenge to healthcare systems as they prepare for the increasing burden of age-related cognitive decline. The prevalence of MCI varies according to several factors, including age, study population, diagnostic criteria, and geographical region. However, estimates suggest that MCI is relatively common among older adults [7]. Several meta-analyses and systematic reviews have examined the prevalence of MCI across different populations and settings. These studies often report prevalence estimates ranging from approximately 10-20% among individuals aged 65 years and older [8]. According to Bai et al., the overall prevalence (affected by age and/or comorbidities) of MCI was 15.56%, the prevalence rates of amnestic MCI were 10.03%, and non-amnestic MCI was 8.72%, while the prevalence of MCI increased with age and decreased with education level [3]. A systematic review and meta-analysis reported a pooled prevalence estimate of MCI of 19.2% among individuals aged 65 years and older in community settings, while the Mayo Clinic Study of Aging found that approximately 16% of individuals aged 70-89 years had MCI [9]. It's important to note that prevalence estimates may vary depending on the criteria used to define MCI (e.g., amnestic vs. non-amnestic, single vs. multiple domains) and the methods used to assess cognitive function. In addition, prevalence rates may be influenced by factors such as population demographics, access to healthcare, and cultural factors [10].

Management strategies for MCI may include lifestyle changes (such as increased physical and cognitive activity) and management of cardiovascular risk factors, aimed at improving cognitive function or slowing progression [11]. Management of MCI often involves addressing underlying risk factors, such as cardiovascular disease, diabetes, or depression, and making lifestyle changes to support cognitive health. This may include regular physical activity, managing stress, and getting adequate sleep [12].

## Review

Methods

The literature was selected to provide comprehensive coverage of the topic from January 2004 to August 2024, using the following keywords: "mild cognitive impairment," "prevalence," "biomarkers," "exercise modalities," "beta-amyloid," "anxiety," "depression," "sleep," and combinations of these in the PubMed and Scopus databases. The studies selected included adult patients, patients with comorbidities, review articles, and meta-analyses, and all articles used were in English. A total of 948 records were identified and 310 studies were considered potentially relevant after an initial screening. After applying the inclusion and exclusion criteria, a total of 35 articles published between 2004 and 2024 were included. The included studies examined the effects of physical exercise on MCI and the subdomains affected by and/or related to MCI.

Pathophysiology of MCI

**Figure 2:**
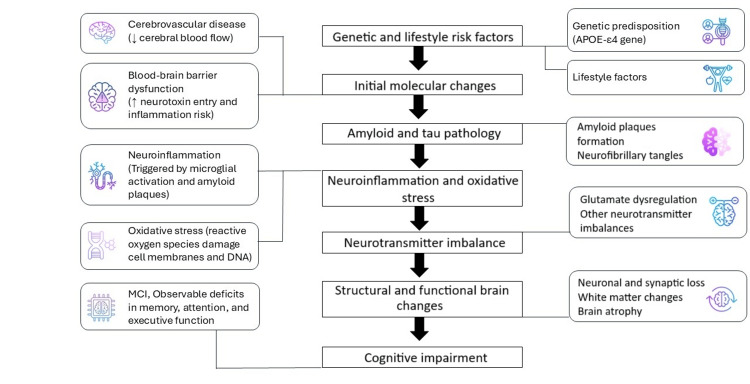
Mild cognitive impairment pathophysiology diagram. Interpretation of the relationship between neurobiological, neurochemical, structural, and vascular changes in the brain that lead to pathophysiological changes and the onset of mild cognitive impairment. The figure is created by the author (Vasileios T. Stavrou) of this study.

Mild cognitive impairment (MCI) is an intermediate stage between normal aging and more severe forms of dementia, characterized by subtle but noticeable changes in cognitive abilities, typically affecting memory, attention, and/or executive functions. The pathophysiological changes in patients with MCI involve a complex interplay of neurobiological, neurochemical, structural, and vascular changes in the brain (Figure 2). The accumulation of beta-amyloid protein fragments that form plaques between neurons is a hallmark of Alzheimer's disease (AD) and is commonly seen in MCI patients. These plaques disrupt cell communication and activate immune responses, leading to inflammation [13]. Chronic inflammation in the brain (neuroinflammation), which involves the activation of microglia (the brain's immune cells), contributes to neuronal damage. Microglial activation can lead to the release of pro-inflammatory cytokines that exacerbate neuronal injury [14]. This leads to blood-brain barrier dysfunction while the integrity of the blood-brain barrier is often compromised in MCI, allowing neurotoxic substances to enter the brain and promote inflammation and neuronal damage [15]. In addition, the abnormal tau protein accumulates inside neurons, forming tangles that disrupt normal neuronal function and contribute to cell death (neurofibrillary tangles). These tangles are often found in regions associated with memory and cognitive function [16]. All of these processes lead to a progressive loss of neurons and synapses, particularly in the hippocampus and cortex, which are critical for memory and cognitive function (neuronal and synaptic loss) [17], while combined with overactivity of glutamate (excitatory neurotransmitter) can lead to excitotoxicity, causing neuronal damage due to glutamate dysregulation, and contributing to cognitive decline [18]. There is also a reduction in acetylcholine (neurotransmitter essential for learning and memory processes), with a deficit similar to that seen in Alzheimer's disease (neurotransmitter imbalance). Changes in other neurotransmitters such as serotonin and norepinephrine may also occur, affecting mood and cognition [19]. With increasing age, a decrease in brain volume, brain atrophy, and white matter changes have been found. Atrophy or shrinkage of brain tissue is seen particularly in the hippocampus, entorhinal cortex, and prefrontal cortex. These areas are critical for memory formation, storage, and executive function, while degeneration of white matter - made up of myelinated nerve fibers that connect different regions of the brain - disrupts communication between these regions [20]. White matter lesions and microvascular damage are common [21]. In addition, certain genetic factors, such as the apolipoprotein E (APOE) ε4 allele, increase the risk of developing MCI and its progression to AD. This allele is associated with the accumulation of amyloid plaques and other pathological processes [22]. Dysregulation of several molecular pathways involved in synaptic function, protein processing, and cell survival may contribute to the development of MCI. These include pathways associated with oxidative stress, mitochondrial dysfunction, and apoptosis [23]. Finally, conditions such as small vessel disease, atherosclerosis, and other forms of cerebrovascular pathology can lead to reduced blood flow to the brain (cerebrovascular disease). This reduction in perfusion can lead to neuronal damage and cognitive impairment. Vascular contributions are significant in a subtype of MCI known as vascular cognitive impairment [24].

Effect of PE on cognition in MCI patients

Emerging evidence from previous studies highlights the complex interplay between PE and cognitive function. Regular exercise promotes the growth and survival of neurons, thereby enhancing neuroplasticity - the brain's ability to reorganize and form new neural connections. This phenomenon is particularly pronounced in brain regions critical for learning and memory, such as the hippocampus [25], while PE affects the production and release of neurotransmitters, including dopamine, serotonin, and brain-derived neurotrophic factor (BDNF), which play a key role in mood regulation, stress resilience, and cognitive function [26]. Exercise also has a positive effect on cardiovascular health, improving blood flow to the brain and strengthening the integrity of the cerebral blood vessels. Optimal vascular function is essential for delivering oxygen and nutrients to brain cells, thereby supporting cognitive processes [27]. In addition, chronic inflammation has been implicated in the pathogenesis of cognitive decline and neurodegenerative diseases, and exercise has anti-inflammatory effects, dampening systemic inflammation and attenuating neuroinflammatory processes in the brain [28]. Finally, mitochondria, the cellular powerhouses responsible for energy production, play a critical role in neuronal function and survival. Exercise promotes mitochondrial biogenesis and function, thereby increasing cellular resistance to oxidative stress and metabolic dysfunction [29].

Effect of PE on biomarkers in MCI patients

Exercise has been shown to reduce the accumulation of beta-amyloid plaques in animal models of Alzheimer's disease. Regular exercise may improve the clearance mechanisms of beta-amyloid, potentially reducing its levels in the brain. Tau pathology, which includes neurofibrillary tangles, can be reduced by exercise [30]. Exercise reduces tau phosphorylation, which is a key process in the formation of neurofibrillary tangles. BDNF is critical for neuronal survival, growth, and plasticity. Exercise increases BDNF levels in the brain, which supports cognitive function and may slow the progression of MCI [26]. Aerobic exercise, such as running or cycling, is particularly effective at boosting BDNF levels [31]. In addition, exercise has anti-inflammatory effects by reducing systemic inflammation, which is associated with cognitive decline, and lowering levels of C-reactive protein (CRP) and interleukin-6 (IL-6). Regular physical activity modulates immune function, reducing the production of pro-inflammatory cytokines [32]. Neurofilament light chain (NfL) is a marker of neuronal damage, and elevated levels are associated with neurodegeneration. Exercise has neuroprotective effects that can reduce NfL levels, promote neuronal health, reduce neurodegenerative processes, and decrease NfL concentrations [33]. Finally, PE improves cardiovascular health, which, in turn, improves cerebral blood flow. Better blood flow can reduce vascular contribution to cognitive impairment, as activities such as brisk walking, swimming, and dancing can improve vascular health, leading to better delivery of nutrients and oxygen to the brain [34].

Effect of PE exercise modalities in MCI patients

Tele-Exercise

Tele-exercise, or remote exercise programs delivered through telecommunications technologies, can be a valuable option for people with MCI. These programs use digital platforms to provide guidance, support, and exercise routines tailored to the individual's needs [35]. Tele-exercise programs remove geographical barriers and allow individuals to access exercise sessions from the comfort of their own homes. This is particularly beneficial for those with mobility limitations or transportation issues. Tele-exercise platforms can provide personalized exercise routines tailored to an individual's physical abilities, cognitive status, and preferences. Some tele-exercise programs include remote monitoring capabilities, allowing trainers to track progress, provide feedback, and adjust the exercise program as needed. By using telecommunications technologies, these programs can provide personalized guidance, support, and motivation from a distance. When implemented effectively and integrated into an individual's care plan, tele-exercise can be an effective tool for managing MCI and improving quality of life [36].

Virtual Reality

Virtual reality (VR) provides immersive environments that can stimulate various cognitive functions, such as memory, attention, and problem-solving skills. Engaging in VR exercises can help maintain and even improve cognitive abilities. Many VR applications combine physical activity with cognitive tasks [37]. For example, VR-based exercise programs can encourage movement that is beneficial to physical health while engaging the brain in spatial navigation, coordination, and multitasking. VR is inherently engaging due to its immersive nature. This can be particularly beneficial for people with MCI who may otherwise find traditional cognitive exercises monotonous or less stimulating. The novelty and interactivity of VR can increase motivation and adherence to regular cognitive training [38]. VR programs can be tailored to the specific needs and abilities of the individual. Customizable difficulty levels and targeted exercises ensure that the cognitive challenges are appropriate and beneficial. VR allows users to practice real-world activities and scenarios in a safe and controlled environment [39]. This can be particularly useful for activities that may be too risky to perform in the real world due to cognitive impairments.

Home-Based Exercise

Home-based exercise programs provide a convenient and accessible approach to promoting physical activity in people with MCI, eliminating the need to travel to a gym or exercise facility and making physical activity more accessible to people with MCI who may have mobility or transportation challenges [40]. Being able to exercise in the comfort and familiarity of your own home can increase adherence to a program. Home exercise programs can be personalized to suit individual abilities, preferences, and safety concerns. Participants can choose exercises that match their fitness level, and modifications can be made as needed to accommodate any physical limitations or health concerns. In addition, these programs offer scheduling flexibility, allowing individuals to exercise at times that are most convenient for them. This flexibility can help overcome barriers such as busy schedules or caring responsibilities, increasing the likelihood of program adherence and being highly cost-effective [41]. Exercise at home can help people with MCI maintain their independence and autonomy by supporting activities of daily living and functional mobility. Regular physical activity can improve strength, balance, and coordination; reduce the risk of falls; and improve overall quality of life. By incorporating exercise into daily activities, people with MCI can reap the benefits of increased physical activity without disrupting their usual schedules. While HBE programs may lack direct supervision, remote support can be provided through virtual platforms, telehealth services, or telephone check-ins [42]. Finally, home-based exercise programs provide a convenient, flexible, and cost-effective way for people with mild cognitive impairment to engage in regular physical activity, which promotes both physical and cognitive health.

Face-to-Face Exercise

Face-to-face exercise ensures that exercises are done correctly and safely. Participation in group exercise programs provides social stimulation, which can improve mood and cognitive function. Socialization is vital for mental health and can reduce the feelings of isolation often associated with MCI [43]. Regular sessions with a trainer or group can increase motivation and adherence to an exercise routine, while instructors can provide immediate feedback. All face-to-face exercise takes place in a structured environment, ensuring that individuals follow a balanced exercise program covering different aspects of physical fitness, including strength, flexibility, balance, and aerobic capacity [43].

Effect of PE on beta-amyloid in MCI patients

The theory of beta-amyloid (Aβ) as the major component of extracellular senile plaques and neurofibrillary tangles in AD and related amyloidopathies involves the production and deposition of Aβ peptides in the brain parenchyma, leading to the formation of neuronal death and ultimately dementia [44]. Exercise can improve clearance of β-amyloid from the brain, reduce plasma concentrations of Aβ42, and reduce Aβ load and amyloid precursor protein metabolism in the brain [45]. This occurs through increased blood flow, improved cerebrovascular function, and increased activity of the glymphatic system, which helps remove metabolic waste, including β-amyloid. Imaging studies using positron emission tomography have shown that patients with MCI who engage in regular aerobic exercise have reduced levels of β-amyloid in regions of the brain associated with Alzheimer's pathology [46]. In addition, exercise-induced reductions in β-amyloid correlate with improvements in memory, executive function, and overall cognition in MCI patients [47].

Effect of PE on anxiety and depression in MCI patients

PE is an important tool for reducing anxiety and depression in people with MCI through neurochemical changes, reducing inflammation, improving sleep, and increasing social engagement. PE can improve mood, reduce stress, and alleviate symptoms of depression and anxiety. Exercise triggers the release of endorphins and serotonin, while exercise increases the production of BDNF, which supports neurogenesis and neural plasticity. Reduced levels of BDNF have been associated with an increased incidence of depressive symptoms, while increased levels of BDNF have been associated with improvements in mood and cognition [48]. In addition, PE is one of the few behaviors that appears to increase neuroplasticity, i.e., the brain's capacity for neurobiological remodeling within key neurocircuits, which is a critical element of adaptive learning, cell growth, and improvement in brain function [49]. PE can reduce depression, lead to changes in brain structure and function, promote hippocampal regeneration, activate prefrontal cortex function, and ultimately improve brain neuroprocessing efficiency and delay cognitive decline [50]. In addition, PE reduces levels of IL-1β and IL-6, and the combination of PE and cognitive training reduces levels of tumor necrosis factor alpha (TNF-α), Αβ40, and total tau, while increasing levels of Αβ42 and Αβ42/40 [51]. Acute exercise promotes a transient increase in pro-inflammatory markers, but long-term exercise may promote a decrease in pro-inflammatory markers and an increase in anti-inflammatory markers through modulatory adaptations, but this depends on anti-depressant medication and comorbidities [52]. PE reduces systemic inflammation and reduces the body's stress response by lowering cortisol levels and cytokines, which can negatively affect brain function and mood [53].

Effect of PE on sleep quality in MCI patients

Sleep is essential for several cognitive processes, including memory consolidation, learning, and emotional regulation, while during sleep the brain performs maintenance functions critical for cognitive health. One of the most important of these functions is the removal of neurotoxic waste products such as beta-amyloid and tau proteins [54]. Inadequate and/or poor sleep quality is associated with a higher risk of developing cognitive impairment. In addition, people with MCI often report sleep disturbances that lead to changes in the brain associated with cognitive impairment, such as disrupted sleep-wake cycles or circadian rhythm disturbances, which are associated with neurodegenerative changes in areas of the brain that regulate sleep [55]. In addition, conditions such as sleep apnea, which is common in older adults, can further exacerbate cognitive decline by causing intermittent hypoxia, which can lead to brain damage and impair cognitive function [56]. Regular PE can lead to improved sleep quality, reduced sleep latency, and better overall sleep quality. Improved sleep, which supports cognitive function and emotional well-being, improves self-efficacy and social engagement [57]. In addition, exercise improves the quality of life and mood in patients with sleep disorders, reduces levels of depression, physical pain, and overall mood disturbance, and increases participation in social activities [58]. PE has positive biological and psychological effects on the brain and cognitive function, promoting a state of well-being, while inducing potent neuroplastic phenomena, partly mediated by epigenetic mechanisms [58].

Effect of PE on well-being in MCI patients

Physical exercise plays an important role in the management of MCI by slowing cognitive decline, promoting brain health, and improving overall well-being. Incorporating regular physical activity into a routine can help maintain cognitive function, delay the progression of MCI to dementia, and improve the quality of life for those affected by cognitive impairment (Table 1). Regular PE improves memory, cognitive and physical function, executive function, balance, coordination, multitasking, cognitive flexibility, and sleep quality, and reduces anxiety, depression, and inflammation [59-63].

**Table 1:** Exercise recommendations for improving cognitive function and managing mild cognitive impairment.

Types of exercise	Example	Duration	Frequency	Intensity	Benefits	Evidence	Study
Aerobic exercise	Walking, jogging, swimming, etc.	150 minutes per week and/or 30 minutes per day for five days a week	3-5 days per week	50-70% of maximum heart rate	Increases blood flow to the brain. Improves memory and executive function. Reduces the risk of progression of the disease	Improves cognitive function and increases the size of the hippocampus	Erickson and Banks [59]
Resistance training	Multi-joints exercise with body weightlifting, resistance band exercises, etc.	45-60 minutes per session, 2-3 sets of each exercise, and 8-12 repetitions per set	2-3 days per week	60-70% of one-repetition maximum	Improves muscular strength and stamina. Improves executive functions such as attention, problem-solving, and working memory	Positive effects on cognitive function, particularly in areas related to executive function and memory	Huang et al. [60]
Balance and coordination exercises	Yoga, Tai Chi, Pilates, etc.	20-30 minutes per session, 2-3 sets of each exercise, and 10-15 repetitions per set	2-3 days per week	Perceived exertion on a scale of 1-10 score, should feel like a 3-5 score (moderate intensity)	Improves balance and coordination. Promotes mindfulness and relaxation	Improve the executive function and processing speed, through mechanisms such as increased brain plasticity and reduced inflammation	Li et al. [61]
Dual-task exercises	Walking while performing a cognitive task, dancing, etc.	30-45 minutes per session, 3-5 sets of each exercise, and 10-15 repetitions per set	2-3 days per week	Perceived exertion on a scale of 1-10 score, should feel like a 4-6 score (moderate intensity)	Challenges the brain and body simultaneously, improving cognitive and physical performance. Improves multitasking and cognitive flexibility	Improves cognitive function compared to single-task exercises by providing a combined mental and physical challenge	Abo et al. [62]
Cognitive-physical combined training	Video games that require physical activity, interactive dance programs	45-60 minutes per session, 3-5 sets of each exercise, and 10-15 repetitions per set	3 days per week	Perceived exertion on a scale of 1-10 score, should feel like a 4-6 score (moderate intensity)	Stimulates the brain and body simultaneously, which can increase cognitive reserve. Increases engagement and motivation through enjoyable activities	Improve cognitive function, particularly in memory and executive function, compared with either type of training alone	Adcock et al. [63]

## Conclusions

Regular exercise is a powerful non-pharmacological tool in the management of MCI. It offers multiple benefits beyond physical health, actively promoting cognitive resilience, enhancing neuroplasticity, and improving brain function. Exercise helps to slow cognitive decline by improving cardiovascular health, reducing neuroinflammation, and promoting the clearance of amyloid plaques. It also contributes to improved mental well-being by reducing stress, anxiety, and depression, which are often associated with cognitive impairment. In addition, exercise improves physical fitness, mobility, and independence, all of which contribute to a better quality of life for people with MCI. Overall, incorporating regular physical activity may be a key factor in maintaining cognitive function and delaying the onset of more severe cognitive disorders.
